# A modified shape context method for shape based object retrieval

**DOI:** 10.1186/2193-1801-3-674

**Published:** 2014-11-15

**Authors:** Radhika Mani Madireddy, Pardha Saradhi Varma Gottumukkala, Potukuchi Dakshina Murthy, Satyanarayana Chittipothula

**Affiliations:** Department of CSE, Pragati Engineering College, Surampalem, AP 533437 India; Department of IT, SRKR Engineering College, Bhimavaram, AP 534204 India; Department of Physics, Jawaharlal Nehru Technological University Kakinada, Kakinada, AP 533003 India; Department of CSE, Jawaharlal Nehru Technological University Kakinada, Kakinada, AP 533003 India

**Keywords:** Object recognition, Shape representation, Feature extraction, Distance measure

## Abstract

The complexity in shape context method and its simplification is addressed. A novel, but simple approach to design shape context method including Fourier Transform for the object recognition is described. Relevance of shape context, an important descriptor for the recognition process is detailed. Inclusion of information regarding all the contour points (with respect to a reference point) in computing the distribution is discussed. Role of similarity checking the procedure details regarding the computation of matching errors through the alignment transform are discussed. Present case of shape context (for each point with respect to the centroid) descriptor is testified for its invariance to translation, rotation and scaling operations. Euclidean distance is used during the similarity matching. Modified shape context based descriptor is experimented over three standard databases. The results evidence the relative efficiency of the modified shape context based descriptor than that reported for other descriptor of concurrent interests.

## Introduction

Although significant progress is witnessed in the field of automated object recognition, it is still remains challenging task (Zhang and Lu 2004; Iyer et al. 2005) from the broad purview of machine learning and computer vision processes of contemporary requirements. The shape of an object contains (Forsyth and Mundy 1999) an important, unique and characteristic features of the object. The shape based methods consider either the contour or the entire region of the object. The consideration of contour involves less representative points in comparison with the region based methods (Nixon and Aguado 2002). The region-based methods consider the global information (all the pixels within a shape) for the design of the descriptor which involves the geometrical moments (Hu 1962; Flusser 2000), Zernike moments (ZM) (Teague 1980; Khotanzad 1990), pseudo- Zernike moments (Belkasim et al. 1991), Legendre moments (Teague 1980), and Tchebichef moments (Mukundan et al. 2001), generic Fourier descriptor (FD) (Zhang and Lu 2002), compounded image descriptor (Li and Lee 2005), shape matrix (Goshtasby 1985), the grid technique (Lu and Sajjanhar 1999) and shock graph (Sebastian et al. 2004; Siddiqi et al. 1999) etc. However, the contour based representation is reported to be more efficient (Yang et al. 2008). Several recently reported contour based methods rely on viz., Fourier transform (Zahn and Roskies 1972; Wallace and Wintz 1980; Kunttu et al. 2006), curvature scale space (CSS) (Mokhtarian and Mackworth 1986; Abbasi et al. 1999, 2000), wavelet transform (Chauang and Kuo 1996; Yadav et al. 2007), contour displacement (Adamek and O’Connor 2004), chain codes (Junding and Xiaosheng 2006), autoregressive (Dubois and Glanz 1986), Delaunay triangulation (Tao and Wi 1999), multi-resolution polygonal (Day et al. 2004) robust symbolic representation (Daliri and Torre 2008), distance sets (Grigorescu and Petkov 2003), elastic matching (Attalla and Siy 2005) etc techniques for the design of the shape descriptor. Basing on the consideration of shape boundaries (Petrakis et al. 2002; Arica and Vural 2003; Bartolini et al. 2005; Lateckia et al. 2005; Alajlan et al. 2007), dynamic programming (DP) technique is also adopted to achieve high accuracy rate. The DP based techniques suffer from being computationally expensive and get reduced to be impractical for large databases, despite the fact that they offer better performance.

Generally, the descriptor relevant to the shape context (Belongie et al. 2002) method for object recognition is developed with an established correspondence between the point sets. The procedure combines the shape context information with the information formatted by using thin plate spline (Bookstein 1989) processing. Due to the proven simplicity and capability of discrimination, the shape context based methods proficiently proposed in the literature (Dubois and Glanz 1986; Tao and Wi 1999; Day et al. 2004; Daliri and Torre 2008; Grigorescu and Petkov 2003; Attalla and Siy 2005; Petrakis et al. 2002; Arica and Vural 2003; Bartolini et al. 2005; Lateckia et al. 2005; Alajlan et al. 2007; Belongie et al. 2002; Bookstein 1989; Mori and Malik 2003; Thayananthan et al. 2003; Zhang and Malik 2003; Salve and Jondhale 2010). Recently, Xin Shu proposed Contour Points Distribution Histogram (CPDH) (Shu and Xiao Jun 2011) for the shape context method. The shape matching process which speaks out the performance of a descriptor is dealt in different ways. The Zucker et al (Siddiqi et al. 1999) has developed shock graph grammar and the relevant tree matching algorithm. The spectral distance (based on diffusion geometry, heat trace) estimated through the Laplacian transform is also used for matching (Bronstein and Kokkinos 2010; Bronstein and Bronstein 2011; Konukoglu et al. 2013). On the other hand, the Fourier transform based matching procedures are is also popular (Cem Direko glu and Nixon 2011; Xingyuan and Zongyu 2013; Ghazal et al. 2009; Ghazal et al. 2012).

In the wake of the results reported in the area of shape context based object recognition techniques involving a wide variety of design of description and matching measures, it serves that the utility of the Fourier based descriptors for the shape context based recognition presents a superior method rather than the contour based methods. Hence, the authors propose for the design of a novel hybrid contour based shape descriptor which is constructed with respect to the centroid, while the feature vector is estimated by a 1D Fourier transform. The shape toning phase is involves the Euclidean Distance to enhance the quality.

The paper is organized in three sections. Introduction to the computerized object recognition method is presented in section-Introduction. Methodology adopted for the present shape context technique is presented in section-Methodology along with the information for indices to evaluate its performance. The results obtained by adopting present method to the standard databases and their trends are presented in section-Results and discussion along with the relevant discussions of performance.

## Methodology

A multi staged novel and hybrid shape context based scheme for the object recognition process is proposed. The phase wise information during the processing is presented in section-Design of system, while the proposed indices to estimate the performance are presented in section-Performance.

### Design of system

**Figure 1:**
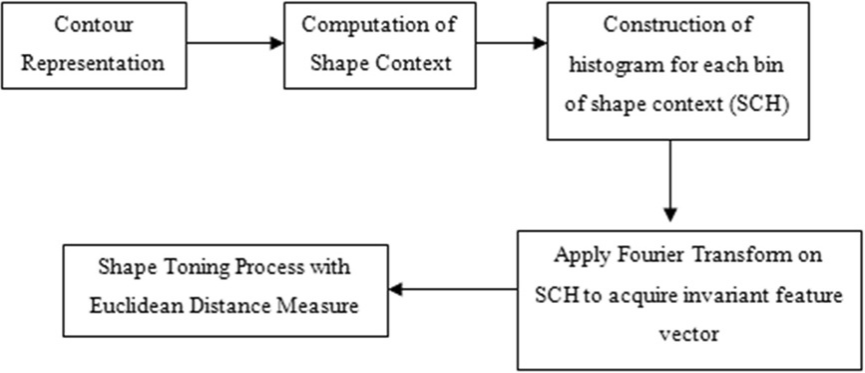
**Schematic diagram of the proposed system with shape context.**

The details of various stages involved with the proposed object recognition by using shape context are schematically depicted in Figure 1. The proposed system consists of four successive steps viz:

(i) **Shape representation with contour**
(ii) **Computation of Shape Context**
(iii) **Construction of histogram for each bin of shape context**
(iv) **Shape description by using Fourier Transform**

**Figure 2:**
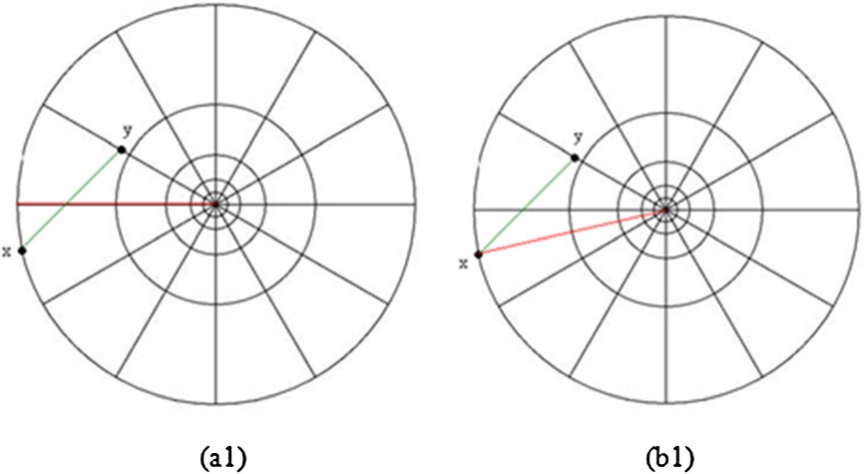
**Example of constructing (a) Belongie Shape Context (b) Modified Shape Context signature for a contour point wrt the corresponding farthest point.**

The descriptor is further expected to a training stage viz., shape toning and ranking. An overview of all these stages of processing implies that the shape based object recognition system includes the salient features of stages, such as shape representation, shape description and shape toning. Contour based shape representation is considered as the initial step of processing. The second step includes description of the shape representation points. Belongie Shape Context (BSC) (Belongie et al. 2002) is popularly used method for describing the shape of the object. During the second step, the contour of the given object is described (Figure 2(a)) by constructing the BSC. During the construction of BSC, the angle between any two points (on log polar transform) is measured with respect to constant center on x axis given by the Equation-(1).
1where:

θ(x, y) is the angle measurement between two points x and y,

y_2_ is the y coordinate of the first point,

y_1_ is the y coordinate of the second point.

To test the invariance property of the BSC, a Modified Shape Context (MSC) is presently proposed (Figure 2(b)). The MSC measures the angle between any two points with respect to the centroid. If the total no. of contour points are Z then the farthest two points will be selected and the angle between these points is measured by the Equation-(2).
2

where:

θ(x,y) is the angle measurement of a point (x, y),

m_1_ is the slope of the line between first point and second point,

m_2_ is the slope of the line between first point and center point.

A histogram containing each bin of the shape context (SCH) is constructed for each part of the shape context to enable the shape context to viable to various transformations. In the wake of the fact that the Fourier Transform is widely used transformations (Ghazal et al. 2009; Zhang and Lu 2005) for object recognition problems and its coefficients are found to be invariant to symmetry operations (i.e. translation, rotation and scaling etc). The size of the shape representation points is an important and influential factor that optimizes the utility of Fourier transformation. Hence, in the shape signature generation process, the sampling is considered as a mandatory step. Some of the sampling methods like Equal Point (EP), Equal Angle (EA) and Equal Arc Length (EAL) (Zhang and Lu 2003) are considered presently. EAL is expected to yield for a better equal space (Peter and Otterloo 1991) than the other two methods. By using EAL, the representation of the contour is restricted to N-number of points. The proposed method uses EAL method to sample the finite number of contour points. For a given contour signal, the 1-D Fourier transform is given as;
3

where:

s(t) represents the 1-D contour signal,

N represents number of representative points of the contour,

n = 0,1,2,…,N-1 and,

FD_n_ represents n^th^ Fourier descriptor.

Using Equation (3), the required Fourier Descriptors of size ‘N’ are generated. Further, the extracted features are testified for their invariance to translation, rotation and scaling operations (performed over the set of images). In the wake of the fact that the proposed descriptor is obtained with respect to the centroid, the obtained features are expected to be invariant to translation. The possible finite (and stipulated) magnitude of the values for the features vouches for the rotation invariance. For the present method, the scaling invariance is also presented by involving the process of dividing the features with the first feature value. In the third step, the feature vector is constructed, which describes the entire shape features of the object.

To further improve the quality of proposed methods, the global information of the object is also considered. For this, experiments are conducted with considering different global descriptors and identified that three global descriptors (GD) are efficient to represent the global shape information. The GD feature vector, viz., {S, C, A} contains the measures of solidity, circularity and aspect ratio is computed for the given object.

In the fourth step, the shape toning process is executed. In the shape toning process, the distance measures (Ghazal et al. 2009, 2012) used is viz., the Euclidean distance (ED). The distance measure between two objects shape context vectors is given by the Equation (4). In this, the average global distance of global feature vectors is directly added to the Euclidean distance of the Fourier descriptor feature vector.

The distance between two shape context vectors including the object global feature vectors is given by the Equation (5).
4

where:
5

ED (TE, TR) represents the Euclidean Distance between the test and trained shapes and,

D_X_ (TE,TR) represents the Global distance between the test and trained shapes.

where:

X represents the GD vector {S, C, AR},

X^TE^ represents the GD feature of the test shape and,

X^TR^ represents the GD feature of the trained shape.

According to the specificity of the data of distance measurement, the distances are further rearranged in ascending order and are assigned with ranks. However, the system is also enabled to recognize and register the top ranked images.

The standard databases (Sikora 2001; Sebastian et al. 2001) used for the evaluation of shape descriptors presently are Kimia {K-99, K-216} and MPEG CE-1 Set B. It is noticed that the Set B database with 70 groups and each group with 20 images. It characteristically includes rotated, scaled, skewed and defected shapes. However the K-99 database which consists of 9 groups, each group with 11 images. It is known to include the partially occluded shapes. The K-216 database with 18 groups, each group with 12 images, it represents a sub database of Set B, and contains partially occluded shapes.

### Performance

The performance of various object recognition schemes reported (Shu and Xiao Jun 2011; Ghazal et al. 2009, 2012) so far employ different measures. Among them, precision and recall are considered as important measures, while they verbally quantify the similarity measurement. Precision (P) and Recall (R) are defined by:
6

where:
7

x denotes the true recognition results,

y denotes the total recognized result and,

P denotes the precision.

where:

R denotes the Recall,

x denotes the true recognition results and,

group size denotes the maximum true recognition result.

The Average Precision value for each recall is computed. This value is affirmatively grouped as two categories viz., Low Recall (LR), High Recall (HR). The Average Precision for Low Recall (APLR) denotes the average precision for recalls less than or equal to 50. In contrast, the Average Precision for High Recall (APHR) represents the average precision for recalls greater than 50. The False Detection Rate (FDR) for each of the image is also estimated by:
8

where:

FDR denotes the False Detection Rate,

z denotes the false recognition result and,

y denote the total recognized result.

The average FDR (AFDR) value of all test images corresponding to each database is estimated. Apart from the usual recognition rate, the Average Processing Time (APT) is also estimated for each query in the shape toning stage. The proposed descriptor is compared with 4 standard descriptors viz., Angular Radial Transform Descriptor (ARTD) (Zhang et al. 2008), Moment Invariant Descriptor (MID) (Zhang et al. 2008), Zernike Moment Descriptor (ZMD) (Tiagrajah and Razeen 2011) and Curvature-Scale-Space-Descriptor (CSSD) (Tiagrajah and Razeen 2011). A specific feature size of 35 for ARTD (n < 3, m < 12), 6 for MID, 34 for ZMD (order from 2 to 10) is adopted. The CSSD feature size is varying from that for one image to another image since number of peaks is varying. All the cited metrics viz., APLR, APHR, AFDR and APT are evaluated to estimate the performance for the proposed descriptor (with inclusion of GD), ARTD, MID, ZMD and CSSD.

## Results and discussion

Shape context based object recognition is estimated as detailed in section-Design of system for the input of standard databases. The trends of the results that follow various approaches are presented in the following sub section-Processing of modified shape context based object recognition. The relative performance of the proposed descriptor is also analyzed in the section-Performance evaluation in the wake of the other reported methods.

### Processing of modified shape context based object recognition

**Figure 3:**
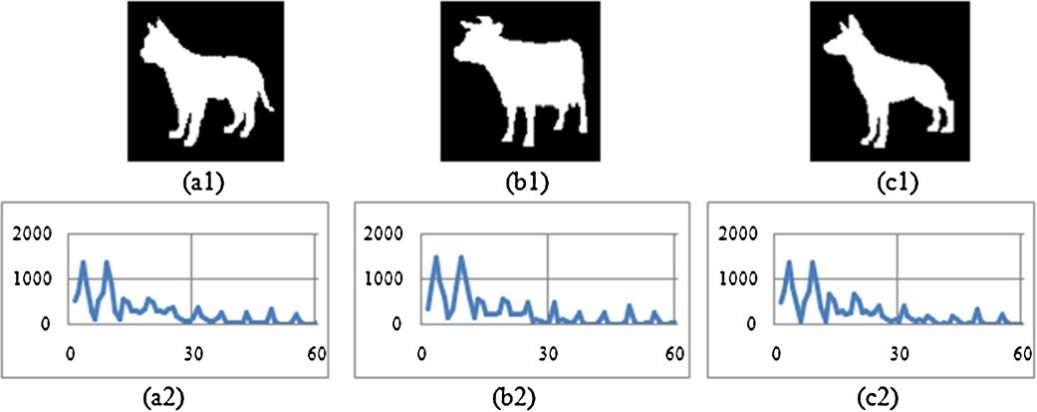
**Proposed descriptor for three Animal group Images (a1) Animal 3 (a2) MSC signature of Animal 3 (b1) Animal 5 (b2) MSC signature of Animal 5 (c1) Animal 7 (c2) MSC signature of Animal 7.**

**Figure 4:**
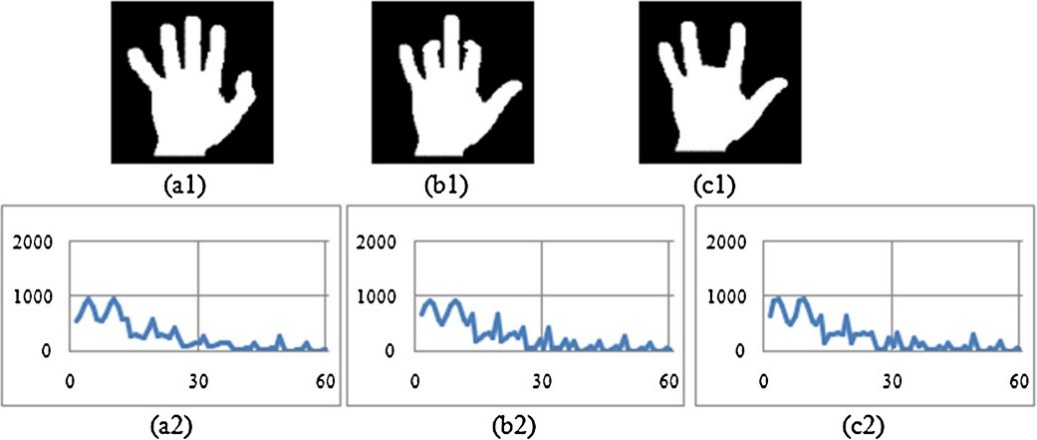
**Proposed descriptor for three Hand group Images (a1) Hand 8 (a2) MSC signature of Hand 8 (b1) Hand 9 (b2) MSC signature of Hand 9 (c1) Hand 11 (c2) MSC signature of Hand 11.**

**Figure 5:**
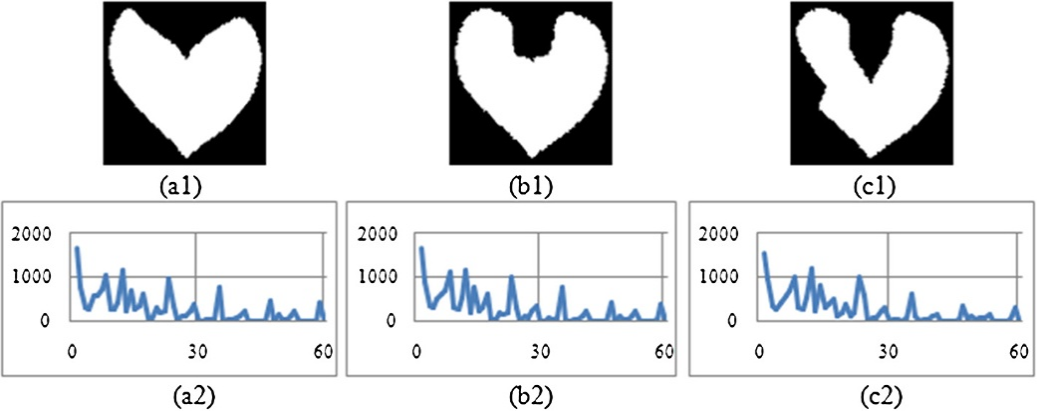
**Proposed descriptor for three Heart group Images (a1) Heart 7 (a2) MSC signature of Heart 7 (b1) Heart 11 (b2) MSC signature of Heart 11 (c1) Heart 12 (c2) MSC signature of heart 12.**

**Figure 6:**
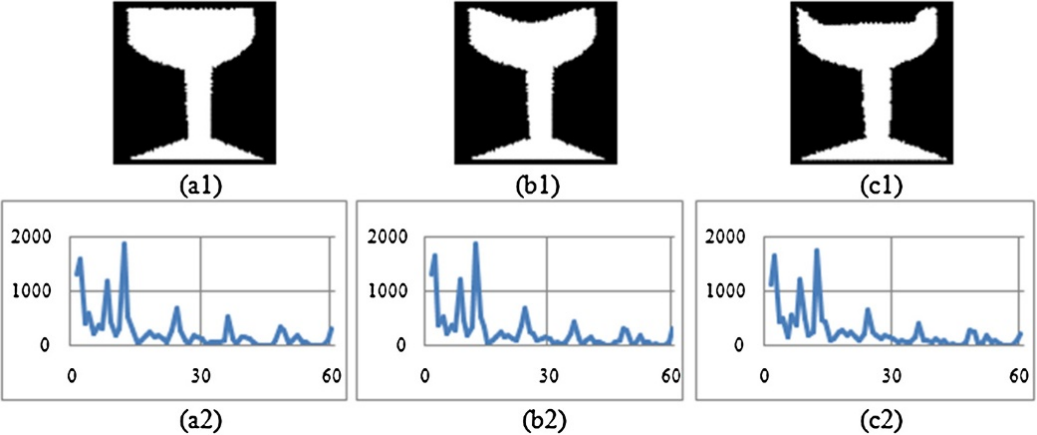
**Proposed descriptor for three Fly group Images (a1) Fly 1 (a2) MSC signature of Fly 1 (b1) Fly 4 (b2) MSC signature of Fly 4 (c1) Fly 10 (c2) MSC signature of Fly 10.**

The shape context is constructed with 60 bins. Then for each contour point, the angle is measured (i.e. BSC and MSC) within the range of one full rotation i.e. from 0° to 360°. A histogram is generated that corresponds to each and every bin of shape context. The histograms, thus constructed are presented in Figures 3, 4, 5, and 6 corresponding to four different image groups (i.e. animal, hand, heart and glass) as accessed from set B, K-99 and K-216 databases. Figures 3(a1), - 3(b1) and - 3(c1) contains three original images of animal group (animal-3, animal-5 and animal-7); the Figures 4(a1), -(b1) and -(c1) pertain to the three original images of hand group (hand8, hand9, hand11); the Figures 5(a1), -(b1) and -(c1) give three original images of heart group (heart-7, heart-11 and heart-12); and Figures 6(a1), -(b1) and -(c1) contain three original images of fly group (Fly1, Fly4 and Fly10). The corresponding Modified shape context (MSC) histogram is illustrated in Figures 3(a2), 4, 5, 6(a2), Figures 3(b2), 4, 5, 6(b2) and Figures 3(c2), 4, 5, 6(c2) respectively. It is clearly noticed from Figures 3, 4, 5, 6, that the MSC histogram is found to be similar for the different shapes within the same group; while it exhibits difference between those of one group compared to the other. Basing on 1-D shape signal, the Fourier descriptors (FD) are generated. The experiments are conducted with varying size of feature vector. From this, it is identified that the first ten features of the FD are consistent. Hence, they are used to design the feature vector.

**Figure 7:**
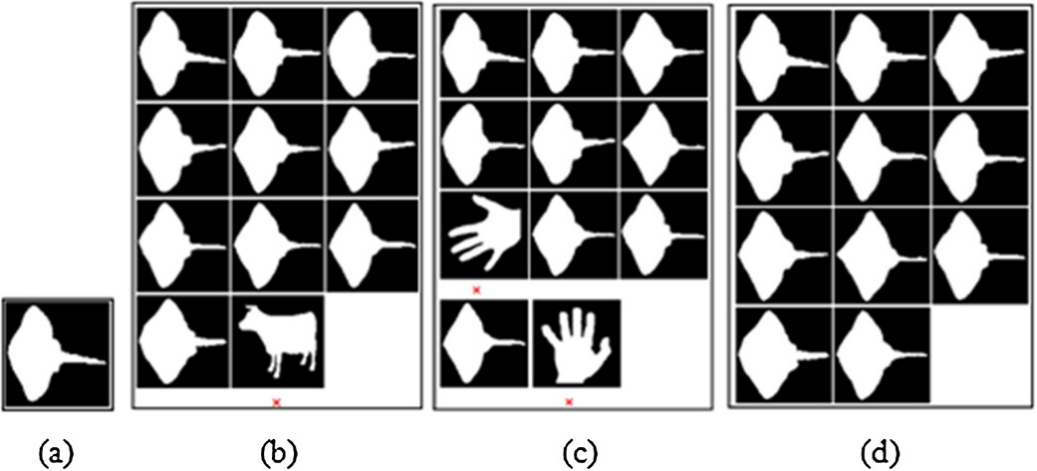
**Retrieval results of Key2 test image from K-99 database (a) Original image (b) ZMD result (c) BSC+GD result (d) MSC+GD result.**

**Figure 8:**
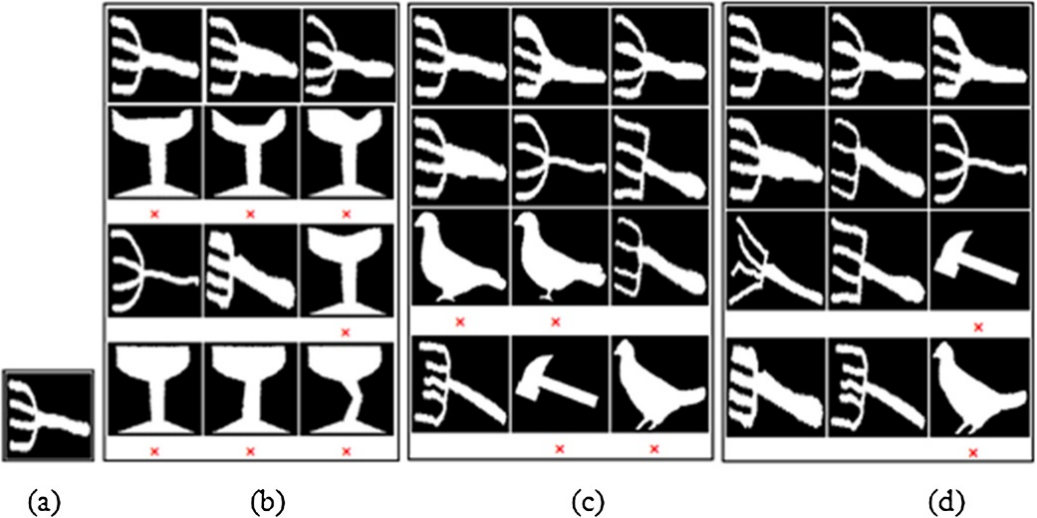
**Retrieval results of Fork12 test image from K-216 database (a) Original image (b) ZMD result (c) BSC+GD result (d) MSC+GD result.**

**Figure 9:**
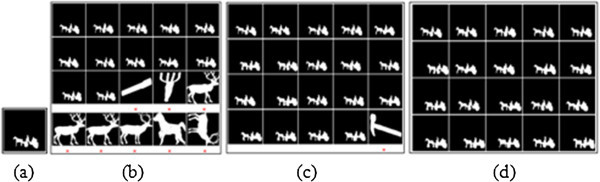
**Retrieval results of Carriage16 test Image from Set B database (a) Original image (b) ZMD result (c) BSC+GD result (d) MSC+GD result.**

In the present object recognition process, Euclidean Distance (ED) measure of performance is estimated between the target and test objects, while they are allocated with ranking according to their distance. In accord with the established procedures, the top n-ranked objects are used to estimate the Precision and Recall parameters. The ‘n’ notifies the group size for {Set B:20, K-99:11 and K-216:12} sets. For each database, the accuracy for the retrieval results corresponding to top ‘n’ (group size of the corresponding database) number of images is estimated and illustrated in Figures 7, 8, 9 respectively. For K-99 database:- The top 11 ranked images correspond to the query image Key2 (as estimated by using ZMD, BSC + GD and MSC + GD), while they are presented in the Figures 7(a)-(d). The Figure 7(a) corresponds to the Key2 query image and Figure 7(b) corresponds to the retrieval results with ZMD descriptor. Figure 7(c) gives the retrieval results for BSC + GD descriptor; and Figure 7(d) vouches for the retrieval results with MSC + GD descriptor. For K-216 database:- The top 12 ranked images corresponding to the query image Fork12 are estimated by using ZMD, BSC + GD and MSC + GD as presented in Figures 8(a)-(d). The Figure 8(a) illustrates the Fork12 query image and Figure 8(b) speaks for the retrieval results with ZMD descriptor. Figure 8(c) gives the retrieval results with BSC + GD descriptor, and Figure 8(d) gives the retrieval results with MSC + GD descriptor.

For Set B database:- the top 20 ranked images corresponds to the query image of Carriage16 by using ZMD, BSC + GD and MSC + GD as presented in the Figures 9(a)-(d). The Figure 9(a) gives the Carriage16 query image, Figure 9(b) gives the retrieval results with ZMD descriptor, Figure 9(c) gives the retrieval results with BSC + GD descriptor and Figure 9(d) gives the retrieval results with MSC + GD descriptor. Overview of cited Figures 7(a), 8, 9(d) suggests that, the MSC + GD descriptor performs better for retrieval of more relevant images with relatively strong correspondence than that with the other descriptors.

### Performance evaluation

The yield of APLR and APHR values for the descriptor with the currently proposed distance measure is analyzed. In the wake of the four other standard descriptors, the aspect of compatibility (with three databases) is also explored, while the results are presented in Tables 1, 2, 3 respectively. From these results, it is clearly evident that the presently proposed descriptor out performs the other descriptors regarding all the three standard databases. However, among the presently considered descriptors, the CSSD descriptor is found to accompany with a lower performance, followed by that of MID, ARTD. However, the case of ZMD resulted for the next higher performance. But, for Set B database, the ZMD is yielding the highest result. From Table 1, it is found that the proposed MSC + GD happen to be influential to increase the APLR value of ZMD. It is also found to significantly increase the APHR value of ARTD. For K-99 and K-216 databases, the ZMD is giving distinctly improved APLR and APHR values than with the other descriptors. From the Tables 2 and 3; it is evident that the proposed MSC + GD is accompanied with an improved performance in terms of enhancement of APLR and APHR.

**Figure 10:**
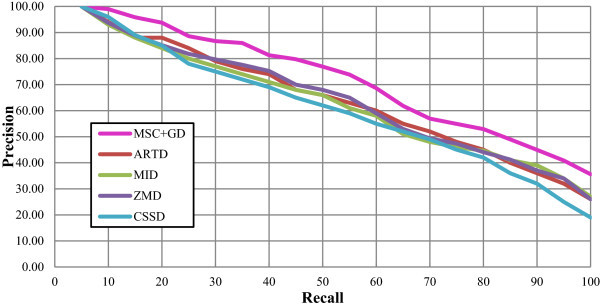
**The PR graph for various descriptors with set B database.**

**Figure 11:**
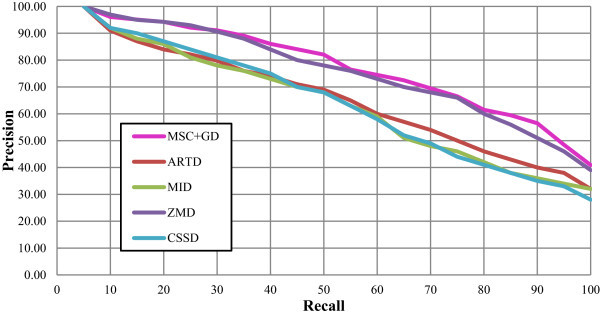
**The PR graph for various descriptors with K-99 database.**

**Figure 12:**
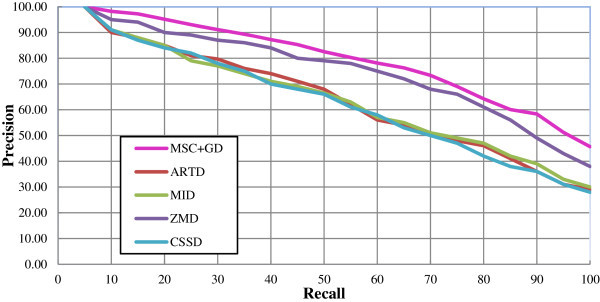
**The PR graph for various descriptors with K-216 database.**

The PR plots for these five descriptors comparing to the set B, K-99 and K-216 are presented in Figures 10, 11 and 12 respectively. Figure 10 reveals that all the five descriptors are yielding considerable enhanced performance with regard to the precision measure for the Set B database in the range of low recalls. At higher recalls, the proposed MSC + GD measure is found to result for improved precision measure, rather than the other standard descriptors. The proposed CSD is found to increase the precision measure marginally at lower recalls i.e. ≤50, bit, it is found to significantly increase the precision at higher recalls i.e. >50. The PR plot for K-99 database is depicted in Figure 11. From this figure, it is observed that the ZMD outperforms other standard descriptors with increased precision measure in the range of both lower and higher recalls. An overview of the results infers that the proposed MSC + GD measure is considerable increase in precision measure at lower recalls ranged between 40 and 50 and higher recalls ranged between 60 to 70 and 90 to 100. The precision measure is found to attain considerable improvement at higher recalls i.e. at 80 to 100. Figure 12 describes the PR plot of various descriptors for K-216 database. The ZMD in this PR plot is found to be superior, rather than other standard descriptors at lower and higher recalls. Thus the proposed MSC + GD measure is giving increased precision measure regarding PR plots at both lower and higher recalls.

**Table 1:** **The APLR and APHR values for various descriptors with Set B database**

	Avg. precision		
Descriptor	Recall < =50%	Recall >50%	Average
BSC	82.65	49.37	66.01
MSC	84.02	49.56	66.79
BSC + GD	84.48	52.85	68.67
MSC + GD	88.83	55.58	72.21
ARTD	82.10	45.69	63.90
MI	79.54	44.50	62.02
ZMD	82.56	45.62	64.09
CSSD	78.61	41.81	60.21

**Table 2:** **The APLR and APHR values for various descriptors with K-99 database**

	Avg. precision		
Descriptor	Recall < =50%	Recall >50%	Average
BSC	86.01	51.25	68.63
MSC	89.11	56.87	72.99
BSC + GD	87.21	57.82	72.52
MSC + GD	90.77	62.43	76.60
ARTD	84.26	45.72	64.99
MI	81.96	44.74	63.35
ZMD	89.61	61.37	75.49
CSSD	82.32	44.11	63.22

**Table 3:** **The APLR and APHR values for various descriptors with K-216 database**

	Avg. precision		
Descriptor	Recall < =50%	Recall >50%	Average
BSC	88.50	57.59	73.05
MSC	90.16	61.18	75.67
BSC + GD	91.01	64.00	77.50
MSC + GD	91.50	66.00	78.75
ARTD	81.35	44.67	63.01
MI	80.14	46.04	63.09
ZMD	88.94	61.71	75.33
CSSD	80.12	44.97	62.55

Other Performance measures viz., Average False Discovery Rate (AFDR) and Average Processing Time (APT) are also estimated as detailed in section-2.2, while the estimated AFDR values for the three databases are presented in Table 4. It is observed that the proposed MSC + GD results for a lower value for all the three databases. Since, the APT measure is argued to exhibit profound influence on shape toning stage (for each of the shape descriptor), it is also estimated for all the three databases, and presented in Table 5. The proposed MSC + GD is found to yield for less APT value in comparison with other descriptors. Therefore, basing the observed trends of performance measures, it is argues that the proposed descriptor exhibits higher efficiency. As a measure of performance, the retrieval rate with bull’s eye score (Cem Direko glu and Nixon 2011) is also estimated. This measure involves the calculation of the ratio of the total number of shapes (i.e. from the same class) to the highest possible number of shapes in the same database. The estimated bull’s eye score for top 40 results in Set B database is presented in Table 6. It is clear that Inner Distance Shape Context (IDSC) is yielding highest score when compared with the others. However, as this includes complicated dynamic programming procedure, the simple Euclidean distance measure for the proposed descriptor is argued to be more efficient retrieval parameter.

**Table 4:** **The AFDR for various descriptors with three databases**

Descriptor	AFDR		
	Set B	K-99	K-216
MSC + GD	0.70	0.72	0.73
ARTD	0.90	0.85	0.86
MID	0.91	0.81	0.84
ZMD	0.78	0.76	0.76
CSSD	0.84	0.88	0.89

**Table 5:** **APT of various descriptors with set B database**

Descriptor	APT
MSC + GD	0.0011
ARTD	0.0017
MID	0.0302
ZMD	0.0017
CSSD	2.1640

**Table 6:** **Bull’s eye score for various descriptors with set B database**

Descriptor	Score %
CSS (Mokhtarian et al. 1997)	75.44
Visual Parts (Latecki and Rolf 2000)	76.45
SC + TPS (Belongie et al. 2002)	76.51
Aligning Curves (Sebastian et al. 2003)	78.16
SSC (Xie et al. 2008)	79.92
CPDH + EMD (Cem Direko glu and Nixon 2011)	76.56
General Model (Tu and Yuille 2004)	80.03
MSC + GD	81.64
IDSC + DP (Ling & Jacobs 2007)	85.40

## Conclusions

- Shape context based description is proved to be efficient when compared with various other standard descriptors with respect to various performance measures viz., APLR, APHR, AFDR and APT.
- The proposed descriptor improves the precision measures at high recalls when compared with the low recalls thus enabling more relevant objects to be recognized.
- With less feature vector size, the proposed descriptor enables the object recognition system to be efficient with less APT and AFDR measures.
